# Tumor-Immune Interaction, Surgical Treatment, and Cancer Recurrence in a Mathematical Model of Melanoma

**DOI:** 10.1371/journal.pcbi.1000362

**Published:** 2009-04-24

**Authors:** Steffen Eikenberry, Craig Thalhauser, Yang Kuang

**Affiliations:** 1 Department of Mathematics and Statistics, Arizona State University, Tempe, Arizona, United States of America; 2 Department of Mathematics, University of California Irvine, Irvine, California, United States of America; University of Washington, United States of America

## Abstract

Malignant melanoma is a cancer of the skin arising in the melanocytes. We present a mathematical model of melanoma invasion into healthy tissue with an immune response. We use this model as a framework with which to investigate primary tumor invasion and treatment by surgical excision. We observe that the presence of immune cells can destroy tumors, hold them to minimal expansion, or, through the production of angiogenic factors, induce tumorigenic expansion. We also find that the tumor–immune system dynamic is critically important in determining the likelihood and extent of tumor regrowth following resection. We find that small metastatic lesions distal to the primary tumor mass can be held to a minimal size via the immune interaction with the larger primary tumor. Numerical experiments further suggest that metastatic disease is optimally suppressed by immune activation when the primary tumor is moderately, rather than minimally, metastatic. Furthermore, satellite lesions can become aggressively tumorigenic upon removal of the primary tumor and its associated immune tissue. This can lead to recurrence where total cancer mass increases more quickly than in primary tumor invasion, representing a clinically more dangerous disease state. These results are in line with clinical case studies involving resection of a primary melanoma followed by recurrence in local metastases.

## Author Summary

Melanoma is a deadly skin cancer that invades into the dermis and metastasizes into the surrounding tissue. In clinical cases, surgical excision of the primary tumor has led to widespread and accelerated growth in metastases. We develop a mathematical model describing the basic process of melanoma invasion, metastatic spread, and the anti-tumor immune response. This model is formulated using partial differential equations that describe the spatial and temporal evolution of a number of different cellular populations, and it uses a realistic skin geometry. Using simulations, we examine the importance of the immune response when a primary tumor is spawning satellite metastases. We find that local metastases can be suppressed by the immune response directed against the primary tumor, but grow aggressively following surgical treatment. We also find that moderately metastatic tumors optimally activate the local immune response against disseminated disease, and in this case tumor excision may have profound effects on metastatic growth. We conclude that surgical perturbation of the immune response controlling local metastases is one mechanism by which cancer can recur. This could have implications as to the appropriate clinical management of melanomas and other solid tumors.

## Introduction

Melanoma, the most dangerous form of skin cancer, arises in the melanocytes and progresses through two well-defined clinical stages. Following a period of radial growth in the epidermis, melanomas may switch to malignant, vertical growth melanoma (VGM) [1]. This switch generally occurs following the onset of angiogenesis and penetration of the basement membrane (BM) separating the dermis and epidermis [2]. These processes are tightly coupled in melanomas [2],[3].

Angiogenesis is induced primarily by the release of angiogenic factors by melanoma cells and associated stromal cells and through the restructuring of the extracellular matrix (ECM) that occurs in concert with invasion [4]. Angiogenesis of lymphatic vessels (lymphangiogenesis) also occurs in melanomas and plays a role in lymphatic metastasis [5]. Melanomas are known to produce a number of angiogenic cytokines, the most prominent being vascular endothelial growth factor (VEGF) and basic fibroblast growth factor (bFGF) [2]. VEGF is overexpressed both constitutively and in response to hypoxia [6].

Although some angiogenesis may occur before penetration of the basement membrane, intense angiogenesis requires ECM remodeling, which in turn requires the cooperation of stromal cells. Even if the tumor is releasing large amounts of VEGF, most of it is sequestered in the ECM [7]. Fibroblast recruitment is essential for the angiogenic switch. Once recruited by platelet derived growth factor (PDGF) and other cytokines, fibroblasts begin producing ECM degrading matrix metalloproteases (MMPs) [8]. Matrix degradation releases large amounts of sequestered angiogenic growth factors, including VEGF, and eases the penetration of new capillaries [7],[8]. MMPs also aid in recruiting stromal cells involved in angiogenesis. MMP-9 induces proliferation and motility of endothelial precursor cells (EPCs) in the bone marrow [8], and MMP-2 recruits VEGF expressing macrophages and leukocytes [9].

Endothelial cells are the predominant cell type in the formation of new vasculature. Endothelial cell migration into the tumoral region is essential for angiogenesis and is facilitated by MMP mediated matrix remodeling [9] and migration up chemotactic and haptotactic gradients [10]. The tumoral vasculature incorporates epithelial cells from the existing vasculature as well as circulating EPCs. The latter are likely important in sustained, intense angiogenesis as they provide a much larger pool of endothelial cells than the native tissue alone can provide [11],[12]. Chemotaxis occurs in response to a number of factors, the most important being VEGF. Growing epithelial sprouts extend filopodia, indicating that they, like epithelial cells, can respond to a VEGF gradient [10].

The immune system is able to effectively mobilize against tumor invasion. This occurs mainly through direct tumoricidal action by natural killer (NK) cells and phagocytes, such as macrophages, and through the T cell response [7]. Unfortunately, the tumor microenvironment is strongly immunosuppressive [13], and while the immune response can hinder invasion, tumor-associated lymphocytes and macrophages have been observed secreting growth and angiogenic factors that may aid in tumor invasion [7],[13],[14].

Melanoma has a strong tendency to metastasize, with most metastases occurring in the skin, lymph nodes, and lungs [5]. Following resection of the primary lesion, cancer can recur locally. This is often due to growth in local satellite metastases and is not caused by incomplete resection [15]. Removal of the primary tumor can stimulate growth in previously dormant metastases [7],[16],[17]. Metastatic melanoma has a very poor clinical prognosis and is largely unresponsive to existing therapies [1].

In this paper we develop a spatially explicit model using partial differential equations (PDEs) to capture the dynamics of melanoma invasion in the skin. We first present a basic model that does not consider an immune response and examine tumor invasion in a cylindrical section of skin. Then we extend this model to include a cellular immune response and carry out a number of numerical experiments using this extended model. In these, we examine the possible dynamics of tumor invasion under different levels of immune response. We develop a method to realistically model the stochastic process of local metastatic spread, and surgical treatment is simulated in both locally metastasizing and non-metastasizing melanomas. These simulations are motivated by a clinical case where, following resection of a primary melanoma, widespread recurrence was seen in local satellite metastases [17].

Based on our simulations we make several observations and predictions. First we observe that angiogenesis is strongly tumorigenic, which is very well known from previous experimental and theoretical work. In accordance with the biological observations in [6], we find that the production of angiogenic factors by melanoma cells in response to hypoxia is insufficient to induce angiogenesis and vertical growth; constitutive production of such factors is needed. These results validate the basic construction of the complex model.

We show that the immune response can either aid or inhibit tumor progression; the outcome depends on the balance between angiogenic factors released by immune cells and the growth-inhibitory and cytotoxic effects of the same immune cells. We also make several predictions concerning treatment and metastasis. We observe that a relatively small safety margin is necessary to remove all primary tumor material and prevent local recurrence due to “local persistence” (as defined in [15]). In the case of a tumor spawning satellite metastases, we predict that the immune response directed against a primary tumor can suppress these metastases “in passing.” The removal of the primary tumor also removes most of this immune response, allowing the cancer to recur through the growth of these previously suppressed metastases. Thus, local recurrence in previously dormant satellite metastases seen in clinical cases [17],[15] can be explained as a consequence of immune disruption.

## Methods

### Mathematical Model

We propose a spatially explicit model of melanoma invasion in the skin, formulated in terms of cell densities. We first present the full system with a detailed derivation of the governing equation for each variable. We then describe the model geometry and boundary conditions.

The basic model considers seven variables:

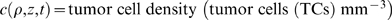

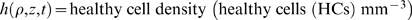

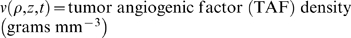

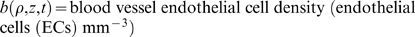

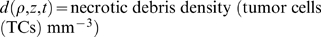

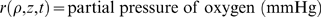

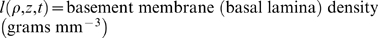

The basic assumptions we use to derive the model follow

1. Cell motility is achieved according to Fick's Law, which yields a diffusion term. Cancer cell motility additionally depends upon contact with other cancer cells and oxygen concentration.
2. Oxygen is the limiting nutrient and diffuses into the skin from the skin surface and a subcutaneous vascular bed [18].
3. Proliferation of both healthy and cancerous cells is mediated by the amount of space available and the concentration of oxygen [19].
4. Cancer cell death is mediated by oxygen concentration, and cancer cells that die in response to hypoxic stress become necrotic debris [19].
5. Cancer cells produce angiogenic factors both constitutively and in response to hypoxia [20],[6].
6. Endothelial cells migrate into the system in response to angiogenic factors and form the tumor vasculature, which supplies additional oxygen [10],[12].
7. A basement membrane separates the epidermis from the dermis and restricts cell migration [2].

The mathematical model is formulated as follows:
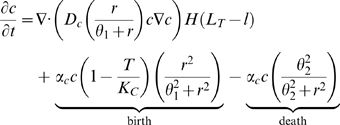
(1)

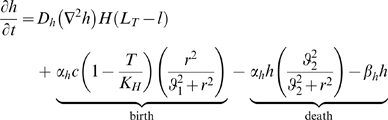
(2)

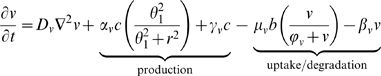
(3)

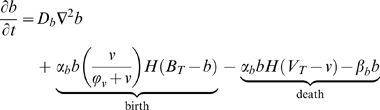
(4)

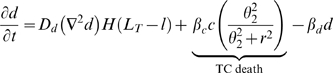
(5)

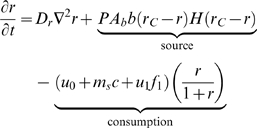
(6)

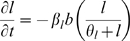
(7)


Where
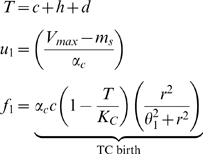



H(x) is the Heaviside step function:
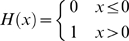



#### Tumor cells

Cancer cell motility is achieved through density-dependent diffusion proportional to oxygen concentration. This is based on the assumption that cancer cell motility requires contact with other malignant cells as well as oxygen sufficient to provide the energy required for migration.

The density dependent term is similar to that used by Tohya *et al.* in a model of basal cell carcinoma [21]. However, in their formulation motility increases linearly with nutrient (i.e. oxygen) concentration. We assume that while low oxygen concentration inhibits mobility, there is little or no benefit to increasing oxygen concentration beyond some sufficiently large concentration. Note that the same parameter, 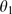, is used to control oxygen dependence for both motility and growth. The coefficient 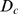 determines how quickly cancer cells diffuse in this fashion and can be considered a general measure of tumor invasiveness and cell motility. For example, loss of keratinocyte mediated control over melanomas would increase 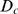, as would an increase in the expression of a number of surface integrins that increase motility and reduce dependence on neighboring cells.

The Heaviside term, 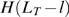, inhibits cancer cell motility in any region where the basement membrane (BM) density is above the threshold 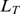. This is biologically well-founded, as cancer cells cannot generally cross the basement membrane unless it is sufficiently degraded.

We assume that cancer cell growth is dependent upon both the local cell density and oxygen concentration. Dependence on density is approximated by a logistic growth term with maximum per capita growth rate 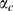. The associated Hill function implies that growth is inhibited when oxygen concentration is low; 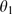 is the partial pressure of oxygen (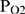) at which half-maximal growth occurs. This Hill function form is widely used in the model, and it has been used in many other models of cancer invasion [22],[23].

We assume that cancer cells also undergo apoptosis in response to extreme hypoxia at maximum rate 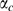. Similar to the term governing growth inhibition, 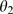 is the 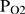 at which half-maximal death occurs. We also assume that malignantly transformed cells suppress normal apoptosis and hence do not experience normal turnover.

#### Healthy cells

Healthy cells are a generic type representing both the epidermal keratinocytes and dermal fibroblasts that make up most of the skin. We assume all healthy cell motility is due to simple diffusion and that cells cannot cross regions of high basement membrane density. Healthy cells grow logistically with carrying capacity 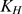 and turn over regularly at rate 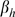. Like cancerous cells, the birth and death of healthy cells is mediated by hypoxia; the growth inhibiting and apoptosis inducing terms are similar to those included in the equation for cancer cells.

#### Tumor Angiogenic Factor (TAF)

TAF can be viewed as an aggregation of those angiogenic factors released by melanomas. The most important of these is VEGF, and wherever possible (e.g. in parametrization) we treat TAF as though it were VEGF. TAF diffuses by simple diffusion and degrades at rate 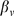. Melanoma cells produce TAF both in response to hypoxia and constitutively. This production varies widely among melanoma strains with the constitutive production typically being the more important source [20],[6]. The maximum rate of TAF expression in response to hypoxia is given by 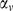, and the Hill function causes this expression to be half-maximal when 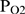 is equal to 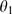. Tumor cells also produce TAF constitutively at rate 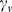. We also include a term representing the uptake of TAF by endothelial cells. This uptake is proportional to the stimulation of ECs by TAF, which is represented by the 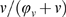 term.

#### Endothelial cells

Endothelial cells (ECs) are the primary cell type involved in capillary construction. We do not explicitly model the tumor vascular network, but instead assume that the density of microvessels is directly proportional to endothelial cell density. Therefore, we only consider the averaged microvessel density and omit the details of capillary construction, which is spatially complex and marked by local irregularities. Since our model considers tumor invasion at the macro-scale, this simplification has a minimal effect on the dynamics.

In this model we assume EC motility is achieved only through simple diffusion. Most models of angiogenesis also consider chemotaxis in response to a TAF gradient [24]. However, in our model geometry an influx of ECs from the vasculature is induced by high TAF concentration. Upon entering the domain, the spatial scale on which they must migrate is only about 1 mm in the vertical domain. Therefore, given the small spatial scale, we conclude that chemotaxis can be neglected in this geometry. Simulations have also confirmed that results are not significantly affected by this omission.

EC proliferation in response to TAF appears to occur mainly when EC density is low. This occurs at the leading edge of a migratory EC front and in existing microvasculature that has been destabilized by TAF to the point that the ECs are pulled away from each other [25]. Therefore, we only allow EC proliferation if the density is below the threshold value 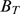. If EC density is below this threshold ECs proliferate in response to TAF. The magnitude of response is controlled by the Hill function 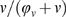, where 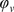 is the value at which half-maximal proliferation is observed. Note that this is the same Hill function as that governing TAF uptake by ECs due to our (implicit) assumption that uptake is translated into growth.

ECs have been observed to undergo apoptosis when VEGF levels are below a certain critical value [25],[26]. Therefore, in the model, instead of proliferating, ECs die at maximum rate 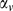 whenever TAF density is below the threshold value 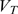. There is also a baseline EC death rate 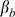.

#### Necrotic debris

Those cancer cells that die become necrotic debris, hence the addition of the cancer cell death term to the right side of the equation. We assume that normal homeostatic mechanisms in the skin prevent healthy cells from contributing significantly to necrotic material. This debris is assumed to disperse at a low rate through simple diffusion and degrade at some small rate 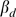.

#### Oxygen

Oxygen diffuses through the skin where it is consumed by both melanomas and normal skin cells, and it is supplied by the tumor vasculature represented by endothelial cell density. Oxygen diffuses by simple diffusion with the diffusion coefficient 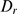. The rate of diffusion varies with skin depth: it is small for the upper epidermis and large for the dermis [27].

The source term represents the supply of oxygen from the tumor vasculature and is modeled according to the principle of solute transport as given in [28]. We consider only diffusion across the capillary and omit any convective transport. Oxygen diffusion is proportional to the 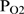 difference between the interstitium and the capillary. The total microvessel surface area corresponding to a single endothelial cell is given by 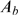, and 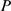 is the capillary permeability coefficient. We make the additional assumption that oxygen is only supplied by, and not lost to, the tumor vasculature, yielding the Heaviside term.

Oxygen is consumed by melanomas and normal skin cells. Here, we include the cancer cell birth term as 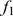. The baseline rate of oxygen consumption in normal skin, 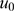, is a constant given by [18]. The baseline rate of oxygen consumption per melanoma cell beyond that consumed by a healthy cell is 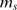, and 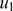 is the oxygen consumed by melanoma proliferation. We use Tristan *et al.* [29] as a guide in deriving this term. Adapting their notation, per capita cell growth 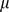 is given as:
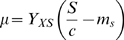
Where 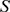 is oxygen consumption, 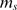 is maintenance oxygen consumption as noted above, and 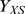 is the yield of cells per unit oxygen, given as:
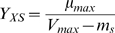


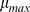
 is the maximum cell growth rate, and 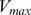 is the maximum oxygen consumption rate. In our model 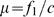, as 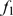 is the total growth for cancer cells and 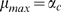. This leads to the following expression for melanoma oxygen consumption:
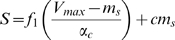
Thus, we arrive at 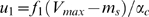.

Finally, if no is oxygen present, then clearly none can be consumed. Therefore, 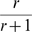 is included to force oxygen consumption to zero if none is present.

#### Basement membrane

The basement membrane (BM) is the most important geometric feature of the skin and has been included. It is a static “wall” across which cancer (and healthy) cells cannot cross. We assume the presence of endothelial cells degrades the basement membrane at rate 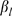; the 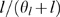 term causes degradation to go to zero as membrane density goes to zero. This term is similar to that used in [23] to represent ECM degradation by cancer cells.

This assumption can be justified biologically as the switch from radial to vertical growth appears to require both the degradation of the BM and angiogenesis, and the two processes are tightly coupled [3]. Migration of ECs degrades the membrane, and matrix metalloproteinases (MMPs) that degrade the membrane must be released along with angiogenic factors to allow EC migration [9]. MMP activity is also necessary to mobilize endothelial precursor cells from the bone marrow [8]. Thus, the EC response to TAF can be thought to encapsulate all mechanisms by which the BM is degraded.

### Geometry

As our model is intended to capture the process of primary melanoma invasion in the skin we must build a reasonable approximation to this geometry. Therefore, we generally consider a three-dimensional domain using cylindrical coordinates – 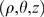. We furthermore assume radial symmetry, eliminating dependence on 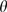 and reducing our consideration to only two independent spatial variables - 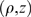.

Considering a cylindrical section of skin, let 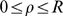 and 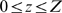, where the z-axis is directed downward from the surface of the skin. We assume that at 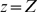 (i.e. the base of the skin segment) there exists a vascular bed. Oxygen is supplied by this vasculature and at the skin surface. Dirichlet boundary conditions are used to hold oxygen concentration constant at the skin surface (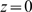) and at the vasculature (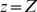). Also, the vascular bed is the source for all endothelial cells entering the skin section. In response to TAF, existing vasculature is destabilized and endothelial cells begin migrating up the TAF gradient. Circulating endothelial precursor cells can also be recruited by TAF and enter the skin from the vascular bed. Therefore, we use a Neumann boundary condition to represent an influx of endothelial cells in response to TAF from both these sources.

**Figure 1:**
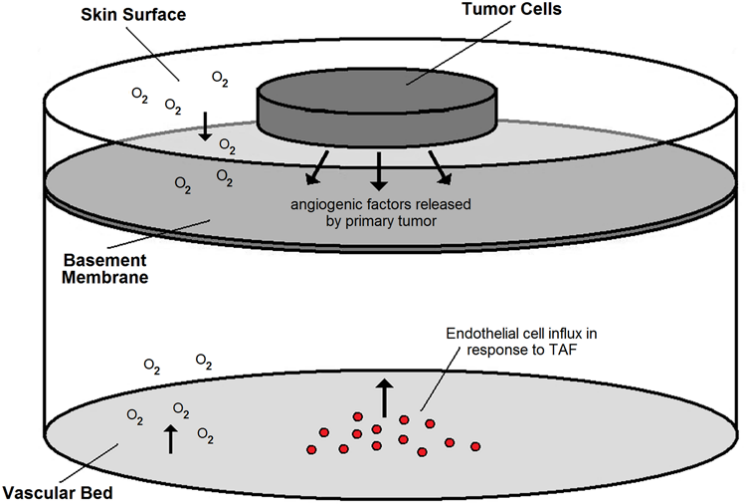
A simple schematic of the modeled geometry. The epidermis is separated from the dermis by a sheet of basement membrane. At the base of the dermis is a vascular bed from which all endothelial cells migrate. Oxygen diffuses into the domain from the vascular bed and at the skin surface. The melanoma tumor originates in the epidermis. All other tissue is initially healthy cells.

Finally, the basement membrane separates the epidermis and dermis, serving as a barrier to migration for most cell types. The basement membrane is modeled using initial conditions in simulations - a thin strip of membrane is thought to exist at a depth of approximately 0.15 mm. A simple schematic of the modeled geometry is shown in Figure 1.

### Boundary Conditions

All boundary conditions are no-flux with several exceptions for oxygen and endothelial cells. The Dirichlet boundary conditions for oxygen are 150 mmHG for the skin surface [18] and 90 mmHg for the vasculature [30]:
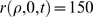


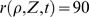



We also provide for an influx of ECs from the vascular bed and circulation through a Neumann boundary condition at 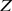 as follows:
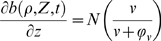



The maximum EC influx is 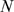, and 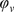 is the TAF concentration at which half-maximal EC stimulation occurs.

### Parametrization for the Basic Model

While this model contains a large number of parameters, we can make at least a reasonable order of magnitude estimate for all of them from empirical biological data.

A wide range of population doubling times has been observed for different melanoma lines, with more advanced tumors typically having higher growth rates. Doubling times range between approximately 1 and 4 days [31],[32]. We always assume that the maximum death rate is roughly the same as the maximum growth rate. The maximum density of melanoma lesions also varies widely. A range from 1.0×10^5^–5.0×10^5^ TC mm^−3^ for several tumor strains is given in [33].

The linear diffusion coefficient for human keratinocytes was measured between .002 and .07 mm^2^ day^−1^ in [34]. Assuming that melanoma cells achieve a similar rate of diffusion at maximum density yields 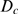 in the range 7.0×10^−7^–4.0×10^−9^, using the range of densities reported in [33].

In [35], a threshold for hypoxia of 10 mmHg is given, with 5–7.5 mmHg considered moderate hypoxia and 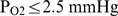 considered severe. We estimate our parameters governing the sensitivity of growth and death to 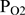 using these values as guidelines. Moreover, we generally assume that healthy cells are more sensitive to hypoxia than cancerous cells. We also assume that production of TAF is proportional to growth inhibition due to hypoxia, i.e. the same parameter, 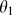, is used in the respective Hill functions.

In a tissue scaffold model the diffusion coefficient for VEGF has been measured as 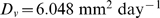 and the degradation rate as 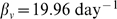 [36]. However, using the relation given in [37] with a molecular weight of 45 kDa for VEGF gives 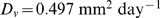, about an order of magnitude lower. Maximum rates of constitutive and hypoxic VEGF expression for several melanoma lines are given in [20],[6].

Using data from [38], the growth rate for bovine endothelial cells is calculated as 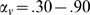. Each endothelial cell corresponds to approximately 5 µ*m* of vessel [22]. Mean vessel diameter for melanoma xenografts varies between 9.5 and 14.6 µm in [39]. This gives 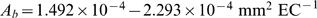 with a mean value of 1.893×10^−4^. Measured values for the capillary permeability coefficient for oxygen are reported to range between 8.64 mm day^−1^ and 6.74×10^4^ mm day^−1^, depending on the tissue type [40].

The baseline oxygen consumption rate, 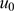, is given in [18]. A number of values for 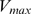 for different cells are given in [18]; we determine a maximum 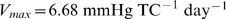.

All parameters and values with references (if applicable) are given in Table 1.

**Table 1:** Parameters and values for the basic model.

Param.	Meaning	Value Range	Ref.
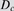	Melanoma density dependent diffusion coeff.	7.0×10^−7^–4.0×10^−9^	[34],[33]
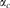	Maximum TC growth rate	.17–.69 day^−1^	[31],[32]
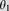	Measures sensitivity of TC growth to 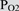	5–10 mmHg	[35]
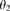	Measures sensitivity of TC death to 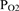	1–5 mmHg	[35]
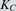	Total cell density above which TCs don't prolif.	1.0×10^5^–5.0×10^5^ TC mm^−3^	[33]
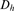	Healthy cell diffusion coefficient	.002–.07 mm^2^ day^−1^	[34]
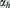	Maximum healthy cell growth rate	.1 day^−1^	
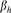	Normal healthy cell turnover rate	.03 day^−1^	
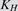	Total cell density above which HCs don't prolif.	1.0×10^5^–5.0×10^5^ HC mm^−3^	
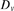	TAF diffusion coefficient	0.497–6.048 mm^2^ day^−1^	[37],[36]
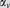	Maximum hypoxic TC expression of TAF	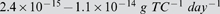	[20],[6]
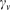	Constitutive TC expression of TAF	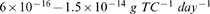	[20],[6]
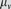	Maximum per capita uptake of TAF by ECs	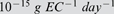	
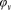	TAF density of half maximal EC response	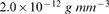	[25]
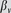	TAF degradation rate	19.96 day^−1^	[36]
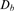	EC diffusion coefficient	.000864–.070848 mm^2^ day^−1^	[42],[43]
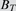	Critical density above which ECs don't prolif.	1000 EC mm^−3^	[48],[49]
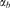	Maximum EC growth/death rate	.30–.90 day^−1^	[38]
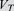	TAF level below which ECs undergo apoptosis	5.0×10^−13^ g mm^−3^	[25],[26]
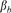	Baseline EC death	.003–.005 day^−1^	[22]
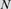	Maximum EC influx	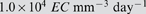	
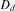	Debris diffusion coefficient	1×10^−6^ mm^2^ day^−1^	
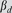	Necrotic debris degradation rate	0.0–.01 day^−1^	
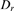	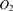 diffusion coefficient	8/85/200 mm^2^ day^−1^	[27]
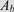	Capillary surface area per EC	1.492×10^−4^–2.293×10^−4^ mm^2^ EC^−1^	[22],[39]
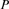	O_2_ capillary permeability coefficient	8.64–6.74×10^4^ mm day^−1^ [40]	
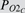	Capillary 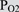	30–40 mmHg	[30]
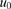	Baseline skin 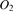 consumption	1.2×10^6^ mmHg g^−1^ day^−1^	[18]
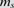	Melanoma maintenance 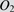 consum. beyond 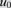	0	[29]
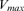	Maximum melanoma 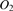 consum. beyond 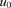	≤6.68 mmHg TC^−1^ day^−1^	[29]
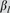	Maximum rate of EC induced BM degradation	1.0×10^−4^ g EC^−1^ day^−1^	
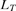	Thresh. BM density above which migration inhibited	.025 g mm^−3^	
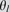	BM density of half-maximal degradation	.01 g mm^−3^	

### Model Extension - Cellular Immune Response

We extend the model to include a class of immune cells that are directly cytotoxic to cancer cells. The most important of these cells are macrophages, dendritic cells (DCs), natural killer cells (NKs), and cytotoxic T cells (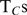). We do not consider the humoral (i.e. antibody generating) immune response.

Melanomas secrete inflammatory cytokines that attract circulating monocytes to the site of invasion. These monocytes can differentiate into macrophages or dendritic cells [13]. Macrophages and DCs are both important phagocytes, capable of engulfing tumor cells as well as fragments of necrotic debris. Macrophages are frequently observed both infiltrating the tumor mass and in the peritumoral region of melanomas. Interestingly, these macrophages can be either harmful or helpful to tumor development. Although macrophages have a cytotoxic effect on tumor cells, tumor-associated macrophages release a number of angiogenic factors, including VEGF, bFGF, IL-8, and 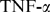 that can aid in tumor progression [41]. In a mouse model by Nesbit *et al.* [14], modest expression of monocyte chemoattractant protein-1 (MCP-1) in melanomas attracted macrophages that proceeded to aid the tumor through release of angiogenic factors. However, high expression led to massive macrophage infiltration that destroyed the tumor within days.

Natural killers do not need any education to recognize and destroy neoplastic cells. On encountering aberrant cells NKs can initiate a large scale immune response through the release of cytokines that recruit other effector immune cells, the most important being tumor necrosis factor 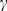 (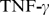) [7].

A strong response by effector immune cells is probably more harmful to the tumor than helpful. The presence of tumor-infiltrating lymphocytes (TILs) has been associated with a good clinical prognosis in a number of cancers. Patient survival is 1.5 to 3 times longer in melanoma patients with high numbers of TILs compared to patients with few TILs [7]. Here we present an extension to the basic model that considers effector cell-tumor interactions. Two new variables are introduced:

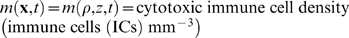

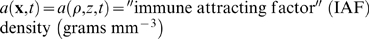

Similar to the TAF formulation, 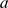 is a generic aggregation of all chemokines that attract macrophages and other immune cells which we refer to as immune attracting factor (IAF). These factors are primarily MCP-1 and 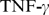. 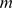 is an aggregation of all cytotoxic effector cells. This aggregate type most closely resembles macrophages and natural killers, as it is cytotoxic to tumor cells and clears necrotic debris. Like natural killer cells, it also has the ability to initiate a larger immune response through secretion of IAF.

**Figure 2:**
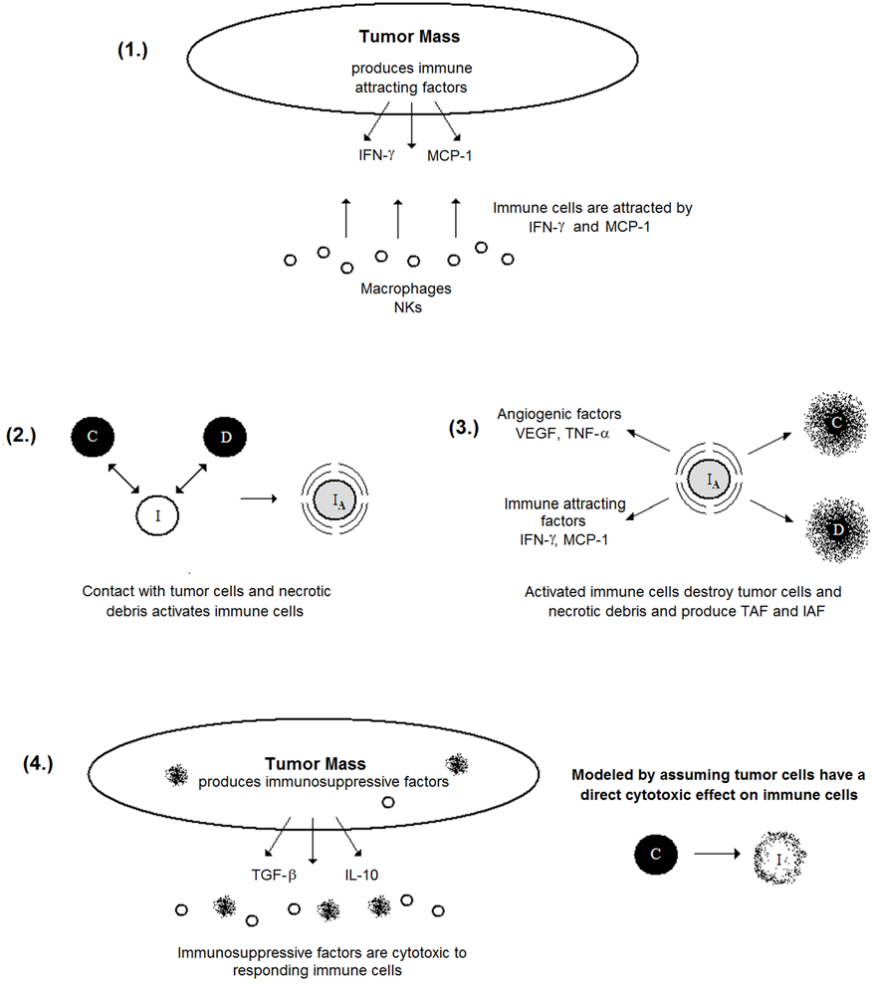
A schematic of the modeled anti-tumor immune response. (1) Cancer cells produce factors that attract immune cells, primarily macrophages and natural killers, to the tumor site. (2) In response to direct contact with either tumor or necrotic cells, immune cells are activated. (3) These activated immune cells kill cancer cells and clear necrotic debris at an increased rate; they also produce both TAF and IAF, enhancing the immune response while potentially inducing angiogenesis. (4) The tumor microenvironment is strongly immunosuppressive, and this is modeled by assuming that direct contact with tumor cells is cytotoxic to immune cells.

We assume that ICs are attracted by IAF and are activated by contact with either tumor cells or necrotic debris. Tumor cells express IAF at a constant constitutive level. ICs express TAF and IAF and have a cytotoxic effect on tumor cells. Contact with tumor and dead cells is assumed to activate ICs. This activation causes immune cells to express both TAF and IAF and increases anti-tumor and debris cytotoxicity. Given that the tumor microenvironment is often immunosuppressive and even directly toxic to immune cells [7],[13], we also assume that tumor cells have a cytotoxic effect on ICs. Additionally, we assume that ICs can devote energy either to debris cleanup or tumor cell destruction. A cartoon of the modeled immune response is shown in Figure 2. The equations governing ICs and IAF, as well as the modifications to the equations for tumor cells, necrotic debris, and TAF, follow (“…” represents the original terms of the equation):
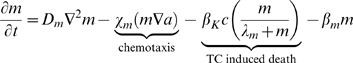
(8)

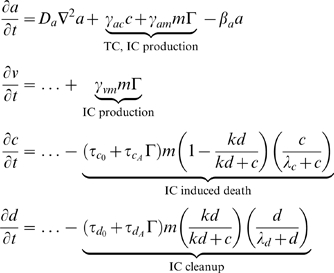
(9)

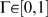
 is the level of activation:
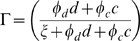
IC mobility is achieved through simple diffusion and a chemotactic response to IAF. 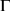 represents the level of activation in response to contact with cancer cells and necrotic debris. 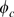 and 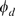 weigh the relative ability of cancer cells and dead cells to activate ICs, and 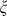 is the (weighted) cell density at which half-maximal activation occurs. 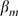 is normal IC turnover. Each cancer cell kills ICs at maximum rate 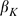. Cancer cells generally do not actually kill immune cells, but if we assume the hostility of the microenvironment is directly proportional to cancer cell density then this formulation is a reasonable approximation. The 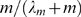 term causes toxicity to saturate with sufficient IC density. Generally, 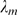 is be taken to be quite small, so that toxicity is at nearly the maximum except at very low IC densities.

ICs are imagined to expend energy at some per capita rate 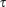 which can be devoted either to debris cleanup or killing cancer cells. The amount of energy devoted to either task is proportional to the presence of debris and tumor cells as well as some inherent bias toward one task or another. Energy devotion to debris cleanup is measured as:
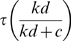


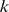
 is a parameter measuring the bias towards debris cleanup. We suppose that 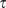 can be scaled in such a way as to measure maximum rate of tumor cell destruction - 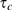, or dead cell destruction - 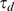. Modifying these further, 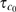 and 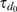 are the respective rates of destruction for a non-activated immune cell. 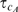 and 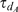 are the the rates of destruction beyond 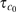 and 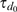 for a fully activated immune cell. Thus, we arrive at the additional death terms for cancer cells: 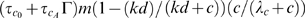. The 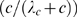 terms cause toxicity to be proportional to cell density, and 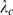 is taken to be very small. The additional death term for necrotic debris is similar.

We assume activated ICs produce TAF at a rate proportional to the level activation, with a maximum of 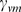. This assumption is justified because, as already noted, tumor-associated macrophages have been observed producing angiogenic factors, and it may be that macrophages that have ingested tumor-derived antigens produce very high levels of VEGF [7].

IAF diffuses by simple diffusion, is produced by tumor cells at the constant rate 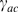, and degrades at rate 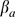. ICs also produce IAF at a rate proportional to the level of activation.

Boundary conditions are no-flux except for IAF. When IAF concentration is high enough we expect an influx of ICs into the domain, representing macrophage, NKs, and 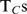 migrating from the circulation. The Neumann boundary condition used follows:
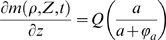



The IAF density at which half-maximal IC migration occurs is given by 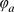, and 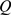 is the maximum influx from the circulation.

### Parametrization for the Immune Extension

We have derived a baseline set of values for most parameters from empirical data. However, a wide range of values is allowed in simulations for those parameters representing cytotoxicity, as these are assumed to be quite variable among strains of melanoma.

We have been unable to find any estimates for the diffusion coefficient for macrophages. However, given that measurements for keratinocytes and endothelial cells fall within the same rough range, .002–.07 [34] and .000864–.070848, respectively [42],[43], we can reasonably assume a similar range for immune cells.

The molecular weight of MCP-1 was measured to be about 4 kDa in [44], although other authors have reported values of 8–10 kDa [45]. Using the relation given in [37] yields 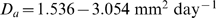. We have been unable to find any estimates on the half-life of MCP-1 either in tissue or in plasma, but we assume it is on the order of several hours. This gives 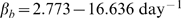 for a half-life of 1–6 hours.

Data on MCP-1 production by melanoma cells is given in [14], from which we calculate 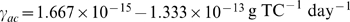. We assume maximum expression by immune cells is similar. We also assume that maximum expression of TAF by immune cells is similar to the constitutive expression by melanoma cells (see [20],[6]).

The turnover rate for human natural killer cells has been measured to be about 2 weeks [46], giving a maximum 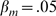. However, since the overall natural killer population remains static, the “effective” turnover rate is probably closer to 0.

To determine the rate at which immune cells kill tumor cells we use data on phagocytosis in macrophages given in [47]. We employ a simple ODE system considering macrophages (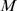), macrophages that have ingested an antigen (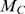), and a tracking variable counting the total number of antigens ingested and processed (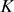).
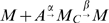
Assuming the antigen level remains constant, this yields the ODE system
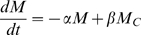
(10)

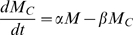
(11)

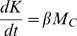
(12)


Solving numerically allows the slope of the kill tracking line (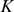) to be calculated, yielding the rate at which macrophages destroy antigen without the need to explicitly model antigen complex populations in the full model. From [47], we estimate 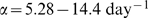 and 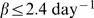, yielding a maximum kill rate of 2.027 day^−1^, presumably for fully activated macrophages. Using 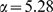 and 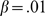, we estimate the maximum rate of destruction for inactivated macrophages to be about an order of magnitude lower, at 0.218 day^−1^.

The parameters 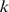, 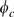, and 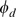 are scaling parameters, included in the formulation for model flexibility. However, for a first approximation we may simply set them all equal to 1. In the absence of other data, and given that macrophage production of MCP-1 is similar to melanoma production of VEGF, we assume that 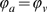.

A tumor cell strain is considered immunoevasive when parameter values are used that give low anti-tumor cytotoxicity and/or a high cell density for activation. A cell strain is considered immunosuppressive if it is highly cytotoxic to immune cells. All immune extension parameters with values and references are given in Table 2.

**Table 2:** All immune extension parameters and values.

Parameter	Meaning	Value Range	Reference
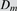	Immune cell diffusion coefficient	.000864–.071 mm^2^ day^−1^	[42],[43]
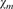	IC chemotaxis coefficient	1.0×10^9^ mm^5^ g^−1^ day^−1^	
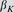	Maximum IC death rate due to tumor cells	.001–.1 IC TC^−1^ mm^−3^	
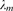	IC density of half-maximal TC induced death	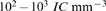	
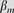	Baseline IC turnover	0.0–.05 day^−1^	[46]
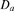	IAF diffusion coefficient	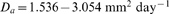	[37],[44],[45]
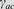	Tumor cell production of IAF	1.667×10^−15^	
		−1.333×10^−13^ g TC^−1^ day^−1^	[14]
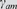	Maximum immune cell production of IAF	1.667×10^−15^	
		−1.333×10^−13^ g TC^−1^ day^−1^	
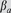	IAF degradation rate	2.773–16.636 day^−1^	
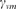	Maximum immune cell production of TAF	0.0–1.5×10^−14^ g IC^−1^ day^−1^	
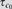	Inactivated IC tumor cell cytotoxicity	≤0.218 TC IC^−1^ day^−1^	[47], see text
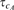	Fully activated IC tumor cell cytotoxicity	≤2.027 TC IC^−1^ day^−1^	[47], see text
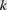	Inherent IC bias towards debris cleanup	1.0–10.0	
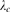	TC density of half-maximal IC cytotoxicity	10^2^–10^3^ TC mm^−3^	
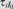	Inactivated IC debris cleanup	≤0.218 TC IC^−1^ day^−1^	[47], see text
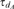	Fully activated IC debris cleanup	≤2.027 TC IC^−1^ day^−1^	[47], see text
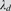	debris density of half-maximal IC cleanup	10^2^–10^3^ TC mm^−3^	
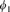	Weighs relative ability of debris to activate ICs	1.0–10.0	
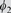	Weighs relative ability of TCs to activate ICs	1.0–10.0	
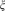	Weighted TC density of half-maximal IC activation	10^2^–10^4^ TC mm^−3^	
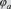	IAF density of half maximal IC response	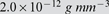	
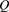	Maximum IC influx	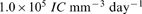	

## Results

### Simulations of the Basic Model

All simulations have been run using a finite difference method on the symmetric cylindrical domain described previously. We run several simulations using the base model without the immune response to characterize the basic dynamics. For biologically realistic parameter values, the model produces realistic patterns of invasion. Before the onset of angiogenesis, growth is restricted to the epidermis. TAF expression by melanoma cells causes an influx of endothelial cells into the domain, which leads to penetration of the basement membrane and vascularization of the tumor within several months. Following this angiogenic switch, density increases and invasion spreads throughout the domain. Live cancer cell density is highest at the skin surface and the vascular bed. Between these boundaries the effects of oxygen consumption by proliferating cancer cells can be seen. Hypoxia is most pronounced near the invasive edge, where oxygen demand is greatest. Necrotic debris is initially concentrated in a roughly spherical core. As the tumor continues to invade, this core expands as an annulus following the invasive edge. Thus, the model predicts that in the absence of an immune response a solid invasive tumor with a necrotic core will form. Angiogenesis is predicted to be strongly tumorigenic.

**Figure 3:**
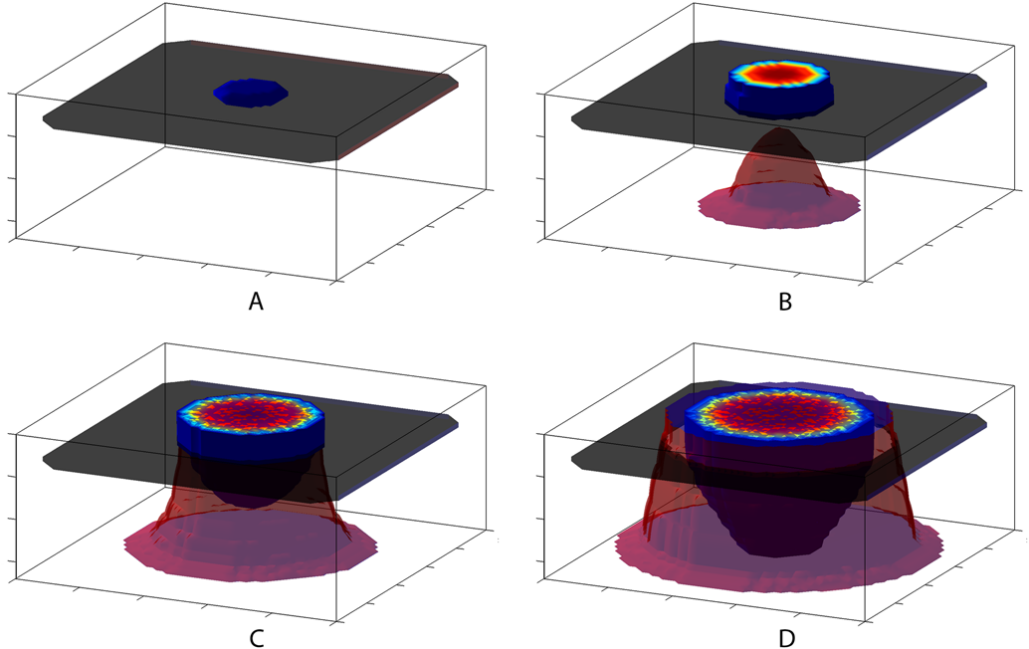
Isosurface of tumor cells (blue), the basement membrane (gray), and endothelial cells (red) for a course of invasion using the basic model. Time steps are evenly spaced over roughly 4.5 months of invasion. (A) An small initial tumor seed in the epidermis. (B) The tumor has spread radially within the epidermis, and endothelial cells have begun migrating toward the tumor. (C) The basement membrane has been penetrated by the endothelial cells, and the cancer cells have begun to invade vertically into the tumor vasculature. (D) A growing vasculature leads the advancing edge of radial invasion. The tumor mass has penetrated to the base of the domain and the vascular bed.

**Figure 4:**
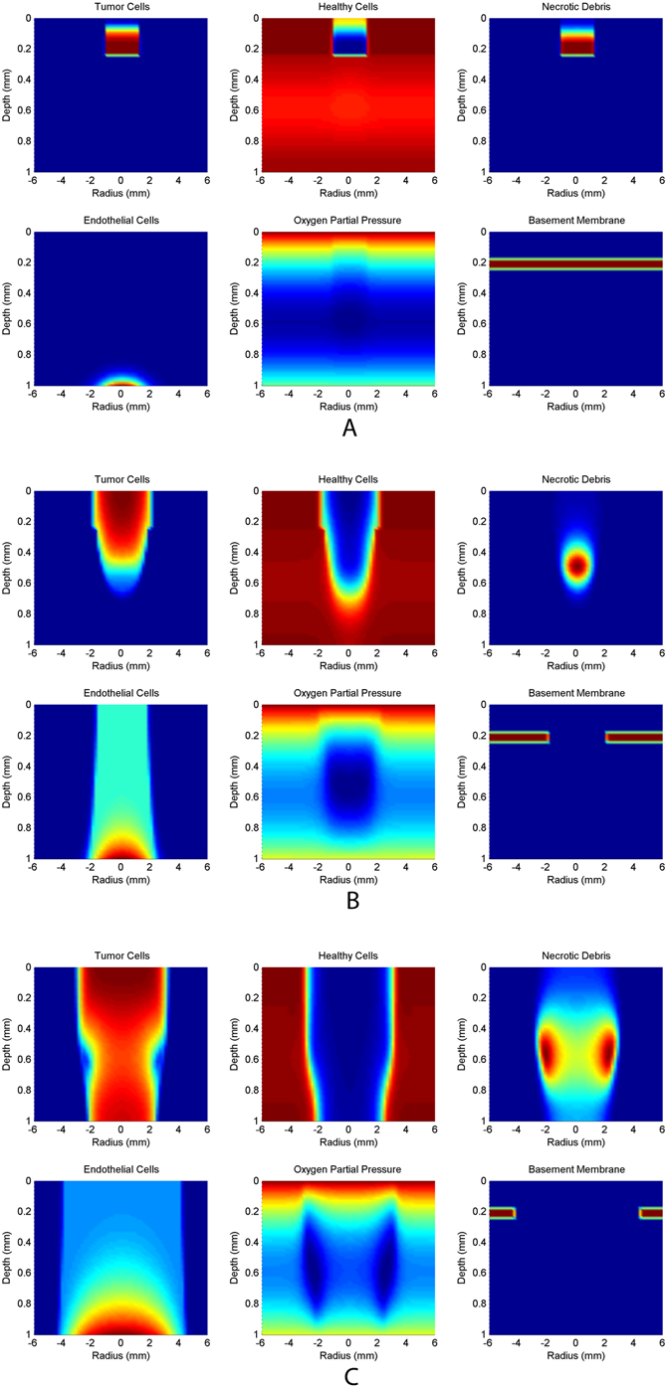
2-D projection of all variables, with the exception of TAF, at three time steps over the course of 6 months of invasion under the basic model. (A) The normal skin is largely intact, but a small epidermal tumor has displaced some of the surrounding healthy cells. (B) The tumor mass has been penetrated by the endothelial cells and, because of the associated degradation of the basement membrane, is invading vertically. The tumor is somewhat hypoxic, a core of necrotic debris has begun to form, and healthy cells continue to be displaced by the tumor. (C) A large tumor has invaded to the base of the domain and continues to expand radially. Hypoxia is most severe at the edge of invasion; this is reflected by the annular expansion of the necrotic core.

**Figure 5:**
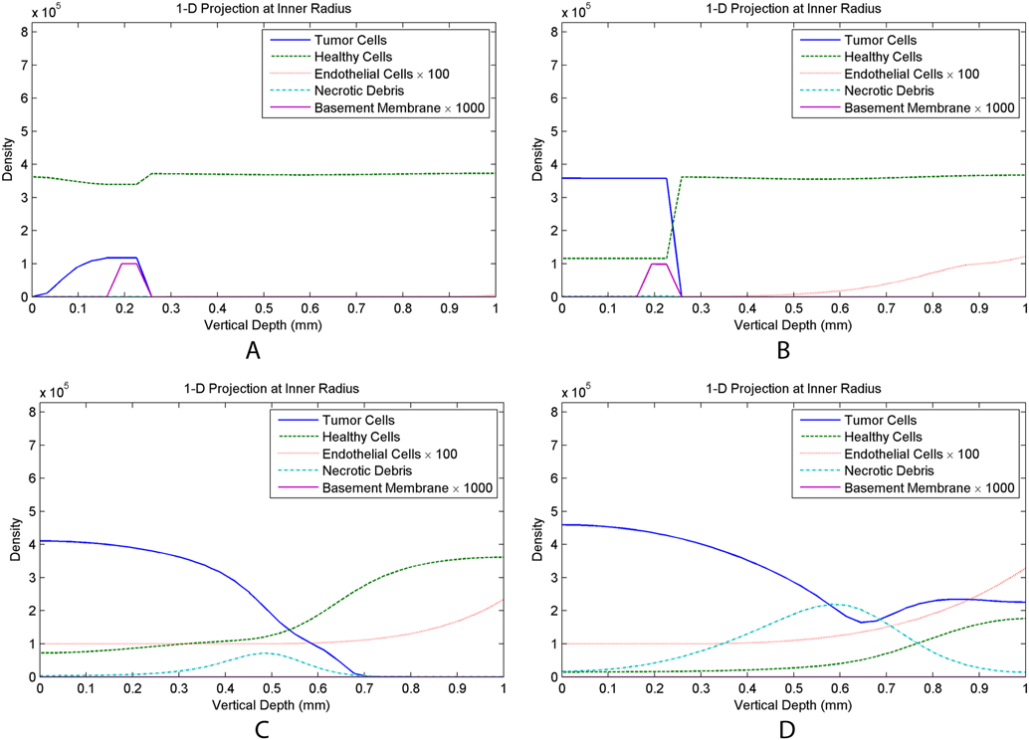
1-D projection of variables densities at the inner radius of the domain. Time steps are evenly spaced over roughly 4.5 months of invasion under the basic model. (A) The seed of a tumor has been planted at the base of the epidermis. (B) The tumor has expanded vertically into the upper epidermis, and has increased in density due to cancer cells out-competing the healthy cells. (C) Here, the basement membrane has been eliminated, and the tumor invades vertically into the existing tumor-associated vasculature. (D) A significant necrotic core has formed roughly in the center of the vertical domain, very few healthy cells remain, and cancer cells continue to grow into the tumor vasculature.

To thoroughly demonstrate the model results, a 3-D isosurface of the evolution of three key variables, cancer cells, basement membrane, and endothelial cells, over several months of invasion is shown in Figure 3. A 2-D projection of the same simulation is shown in Figure 4. Finally, a 1-D projection of variable densities at the inner radius of the domain is shown in Figure 5.

A second set of simulations is performed in which the cancer cell line does not produce TAF constitutively. In these simulations, TAF production in response to hypoxia is not sufficient to induce angiogenesis, and tumor growth is restricted to the epidermis. Even when TAF production in response to hypoxia is set to the highest value reported in [6] and maximum tumor cell density is used, angiogenesis does not occur and growth remains radial. If the same simulations are run with no hypoxic TAF production but with the lowest rate of constitutive TAF production reported in [6] then angiogenesis occurs, leading to an aggressive, vertically invasive tumor. Therefore, we predict that the constitutive production of TAF is much more important in melanoma of the skin. This is in line with the conclusions made by Danielsen and Rofstad in [6]. However, we note that constitutive production of TAF by melanoma cells themselves may still be be unnecessary, as stromal cells play a very important role in producing the angiogenic factors and MMPs necessary for invasion, as discussed previously. We can therefore conclude that TAF production in response to hypoxia is insufficient to induce angiogenesis - TAF must be produced by melanoma cells constitutively or supplied by cooperating stromal cells.

### Immune Response

In simulations of primary tumor invasion with an immune response, tumor growth is generally slowed significantly. There appear to be three possible eventual outcomes:

1. The tumor is completely destroyed.
2. A pseudo steady state is reached where the tumor ceases growing, and immune cell infiltration levels remain constant.
3. The tumor succeeds in completely invading the domain.

All three outcomes occur in biologically reasonable parameter space. The pattern of immune cell infiltration differs between tumors that can be characterized as immunoevasive versus immunosuppressive. In immunoevasive tumors, immune cell levels are high in the core, while suppressive tumors result in a high peritumoral concentration with little core presence.

**Figure 6:**
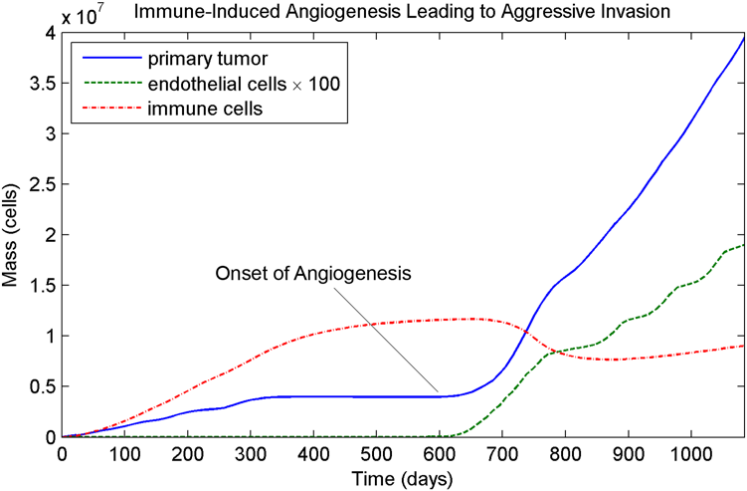
Two-phase pattern of invasion where the immune system holds tumor growth to an initial steady state in the epidermis. This steady state persists for about 300 days, but the expression of angiogenic factors by activated immune cells induces angiogenesis that leads to aggressive, unchecked tumor invasion.

We have also examined the effect of TAF in expression by activated immune cells. This can cause a two-phase pattern of growth, where the immune response initially holds the tumor to a steady state within the epidermis. After a period of apparent quiescence, immune-induced angiogenesis leads to a second phase of more aggressive vertical growth. Surprisingly, the tumor can completely invade the domain in this second phase even if the immune response was sufficient to hold it to a steady state in the first phase. The results of such a simulation are shown in Figure 6.

### Surgical Treatment

To simulate treatment, the tumor is allowed to grow for some specified amount of time, after which surgical excision is performed. To simulate excision the value of all variables is set to zero within a prescribed region. Then the simulation is allowed to continue for several years.

The results of a surgical excision using the basic model without an immune response are simply characterized. Immediately following excision the wound is quickly filled by healthy cells, and in the following absence of TAF what remains of the tumoral vasculature quickly dies off. However, within a year any surviving tumor cells begin invading again, and soon the tumor recovers to pre-surgery mass.

In simulations without an immune response the tumor border is typically sharply defined with little spread beyond the visible border. A margin of several millimeters beyond the visible edge of the tumor is generally sufficient to ensure no tumor cells survive.

### Surgical Treatment in a Metastasizing Tumor with Immune Response

To simulate a primary tumor seeding metastases in the nearby tissue a normal simulation is first run for some initial amount of time (typically 6 months). Then a very small metastasis is introduced some distance from the primary tumor at the level of the vasculature. Note that this metastasis is necessarily a “metastasis ring,” due to the symmetry of the domain. This is not an unreasonable approximation, as metastasis spread is be expected to be roughly symmetric, and a complete ring can be thought to represent a worst-case scenario.

We assume that metastasis seeding can be modeled as a Poisson process, i.e. the probability of a metastasis being created within a given time period is an exponential random variable. We furthermore assume that the distance from the primary tumor at which the metastasis is seeded is exponentially distributed, with the greatest probability next to the tumor edge. Thus, a biologically reasonable approximation of metastatic spread into the surrounding skin tissue can be described by two exponential rate parameters, 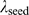 and 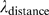.

To explicitly differentiate between primary tumor and metastasis populations an additional cancer cell variable is introduced. A number of minor modifications must be made to the model; they are straightforward and we do not present the details.

**Figure 7:**
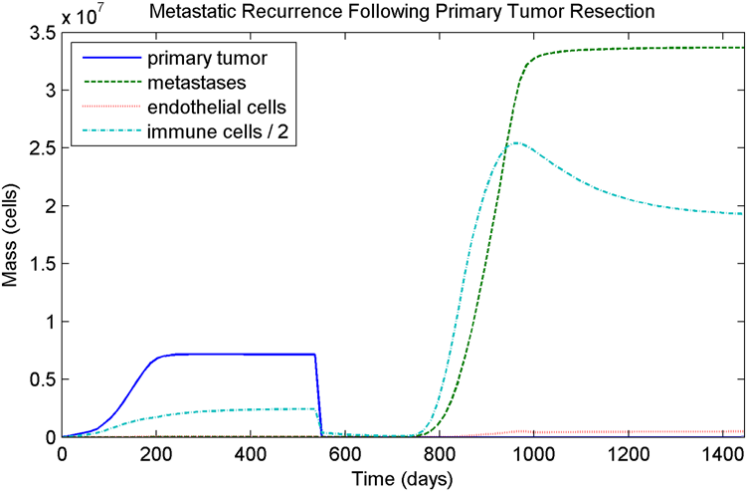
Resection followed by metastatic recurrence. The primary tumor is held to a steady state and metastases are present at undetectable levels prior to resection. Following resection, aggressive metastatic recurrence occurs. The asymptotic behavior of the metastases is the same as the primary tumor (i.e. steady state), but the overall growth rate is faster, and the final cancer load is much greater.

**Figure 8:**
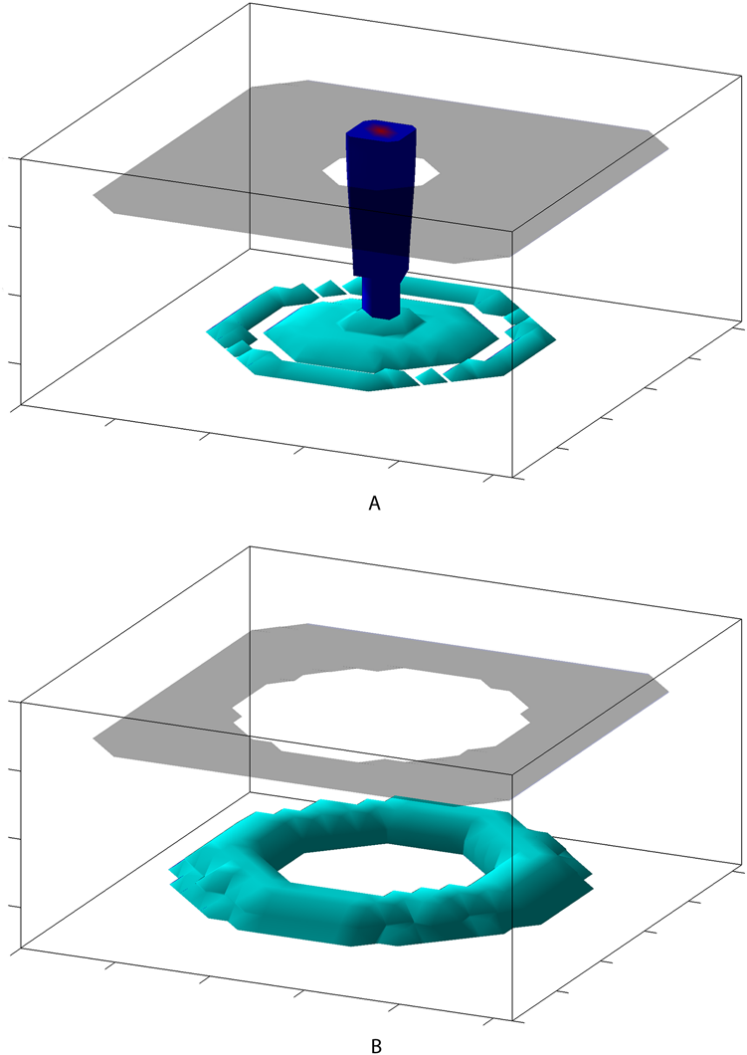
Isosurfaces of a metastatic primary tumor before and after resection. Primary tumor (blue), metastases (cyan), and the basement membrane (gray) are shown. (A) The primary tumor at a steady state along with suppressed metastases. The exact picture shown is representative of the fluctuating metastatic load, which varies somewhat, although the overall behavior is one of a quasi-steady state. (B) Metastatic recurrence 6 months after resection of the primary tumor. The resection was successful in removing all material from the primary tumor, but the total mass of the metastases already exceeds that of the primary tumor.

The immune response is included in these simulations. If no treatment is performed the primary tumor invades normally while the metastases, if sufficiently close to the primary tumor, are generally destroyed or held to an extremely low density. We have found that if 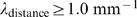, then all metastases are typically suppressed. If the primary tumor is removed, any surviving metastases are often able to begin invading. The immune response eventually reacts to the metastases, and the same asymptotic behavior occurs (a steady-state is reached or the domain is invaded). However, the metastases often increase in total mass much more quickly than did the primary tumor. Such a course of treatment followed by metastatic recurrence is shown in Figures 7 and 8.

**Figure 9:**
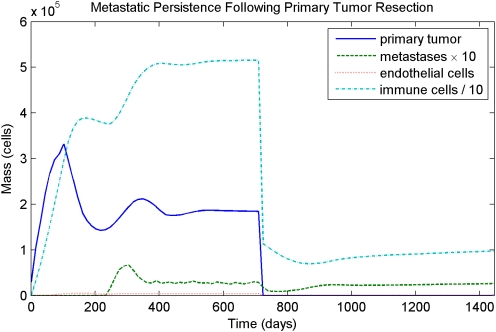
Resection of a strongly immunogenic tumor followed by low-level metastatic persistence. In this case, the immune response holds the metastatic load to a total mass much less than that of the primary tumor, and unlike in the cases shown in Figures 7 and 8, resection of the primary tumor does not induce growth in these metastases. However, a small but biologically relevant metastatic load remains, suggesting that dormant metastases can persist nearly indefinitely and may be sensitive to future perturbations of the system.

Resection can lead to aggressive metastatic recurrence, but in some cases where the immune response is very strong, resection can lead to a state where metastases persist but remain held to a small size. This state of persistence can last indefinitely, and an example is shown in Figure 9.

**Figure 10:**
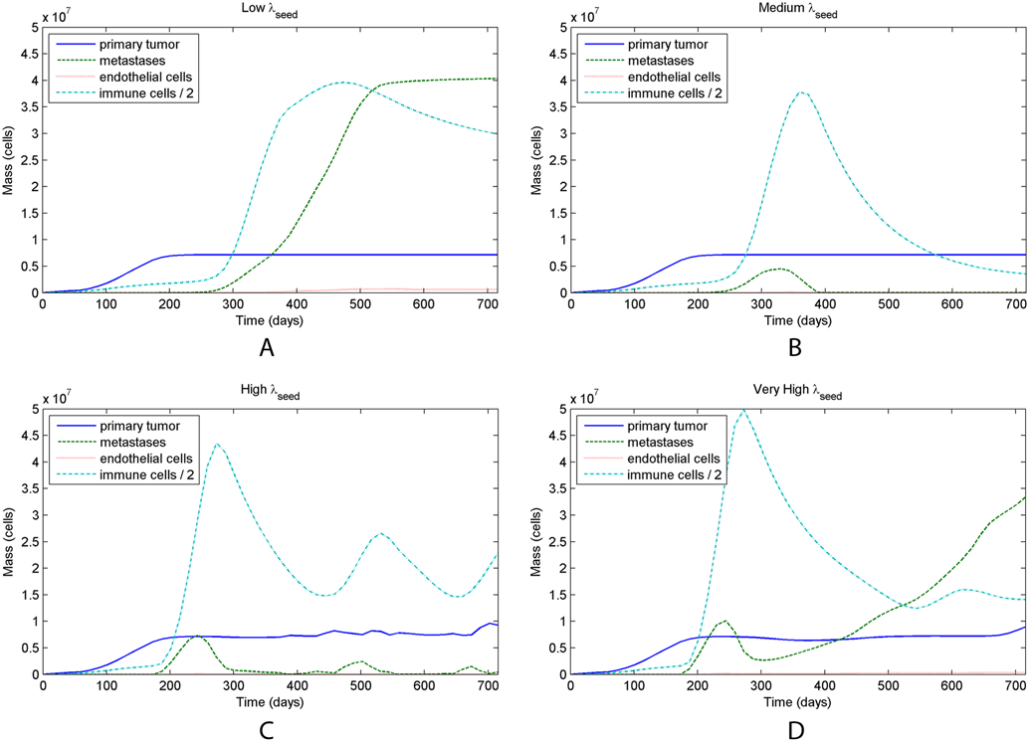
Metastatic growth under different values of 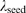. The primary tumor is held to a steady state by the immune response, and metastases are seeded beginning 6 months into the simulation. For the lowest value of 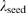, a single metastatic lesion forms; moderate to high values lead to suppression of all metastases, while a very high 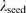 overwhelms the immune response, leading to widespread metastatic growth. Note that in (C) and (D) the immune response to the metastases can cause perturbations in the primary tumor size, but does not change the overall behavior. (A) 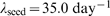. (B) 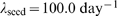. (C) 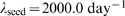. (D) 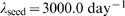.

Numerical investigation has yielded a somewhat nonintuitive result concerning the rate at which metastases are seeded. In a sensitivity analysis of 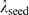, we have found that for low values of 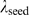 a single secondary metastasis usually forms. Upon increasing 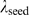 to fairly large values, significant metastatic spread occurs, but it is eliminated by the immune response. Very large values of 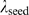 cause metastatic disease to overwhelm the immune response and invade widely. We note that the initial secondary metastasis generally occurs at a predictable distance from the primary tumor, just beyond the range in which the immune response directed against the primary tumor can incidentally suppress metastases. Figure 10 shows the results under several values of 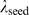.

These results suggest that moderately aggressive primary tumors that seed many metastases can induce a widespread local immune response that is sufficient to keep these metastases in check. Furthermore, those metastases that do manage to grow to significant sizes are quickly eliminated by the immune response, even though this response cannot eliminate the primary tumor.

## Discussion

We have examined the macroscopic dynamics of melanoma tumor growth using a reaction-diffusion framework. This diffusion framework models cell populations as a continuous density field, and has the effect of washing out local inhomogeneities. However, tumors have an irregular and heterogeneous architecture, and angiogenesis in particular is a spatially complex phenomenon, with the tumor vasculature marked by irregular construction and heterogeneity in blood flow [19]. Local perturbations and substructures in the vasculature likely have a minimal effect on the tumor's overall growth and architecture.

Of more concern is the variability in nutrient supply, principally oxygen, caused by a regionally heterogeneous vasculature, and its potential effect on overall tumor growth. However, our model suggests that melanoma is rather unique among solid tumors, with significant hypoxia only occurring at the leading edge of invasion. This is due in large part to the significant amounts of oxygen diffusing into the tumor core from the skin surface. Thus, the unique geometry in which melanoma invades likely dampens the importance of vascular irregularity. Therefore, we argue that the diffusion approximation can reasonably be employed in examining melanoma tumor invasion at the macroscopic level.

Using this model as a framework for early investigation, we have observed a wide range of interesting and biologically reasonable patterns of tumor invasion. Angiogenesis is strongly tumorigenic in this model. In simulations using the basic model, following the onset of angiogenesis the tumor spreads throughout the dermis and a significant necrotic core forms. Hypoxia is always most severe at the leading edge of radial invasion, and the necrotic core expands as an annulus in sustained invasion. The constitutive production of TAF (particularly VEGF) is more important than production in response to hypoxia in inducing angiogenesis in melanomas. In simulations, even the most aggressive cancer cell strains are unable to induce angiogenesis without at least a low level of constitutive TAF production. Therefore, TAF must be produced constitutively by melanomas or by cooperating stromal cells.

When an immune response is considered, it usually inhibits tumor growth, often destroying invasive tumors or holding them at a steady state for many years. These outcomes are observed in biologically reasonable parameter space, implying the immune response often plays a clinically meaningful role in the control of cancer growth. However, immune cells expressing TAF can also aid melanoma invasion by inducing tumorigenic angiogenesis. This can lead to a qualitative change in tumor behavior as non-invasive melanoma tumors restricted to the epidermis become aggressively invasive following immune induced angiogenesis.

We have investigated a primary tumor seeding micro-metastases into the local skin tissue. This line investigation is motivated by the case study reported by De Giorgi *et al.* in [17], where a patient presented with a large polypoid melanoma lesion that had reportedly been growing slowly for about three years. Following resection there was rapid recurrence in a number of previously dormant satellite metastases within 5–7 cm of the original lesion. This case was previously examined in a mathematical model of tumor dormancy by Boushaba *et al.* [16]. This is not an isolated case, as many cases of local melanoma recurrence are actually caused by local micro-metastases and not by incomplete resection of the primary tumor [15].

We propose that local metastatic spread can be reasonably modeled using two exponential rate parameters. The first, 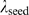, gives the rate at which micrometastases shed from the primary tumor into the circulation. The second, 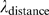, determines the distance from the primary tumor at which extravasation occurs. Under this model, simulations have suggested that moderately metastatic melanomas may induce local immune activation that is optimal for the suppression of metastatic disease. Melanomas that seed metastases only occasionally do not induce sufficient immune activity to destroy metastases beyond a threshold distance, while extremely metastatic melanomas overwhelm the immune response.

This framework also suggests an explanation for the phenomenon of aggressive metastasis growth following surgical excision of a primary tumor. The immune response directed against a primary tumor can suppress nearby metastases. Following surgical excision most of the immune cells attacking the primary tumor are removed, as is the major source of cytokines attracting other immune cells to the sight. In the absence of this immune response, previously checked metastases can begin growing aggressively. The total mass and growth rate of these metastases can exceed that of the primary lesion, making this recurrence potentially more clinically dangerous. This phenomenon was also studied in a mathematical model by Boushaba *et al.* [16] who found that the release of growth inhibiting cytokines produced by the primary tumor is a possible mechanism. Anti-angiogenic factors released by the primary tumor, such as angiostatin and endostatin, are another major explanation [17]. Metastasis suppression by these factors and the immune response are not mutually exclusive hypotheses, and all may play a role.

There is clearly a threshold distance beyond which local immune activation is insufficient to suppress metastases. This distance may be highest in moderately metastatic tumors. It is possible that immune activation plays the dominant role in suppression near the primary tumor, where most metastases are expected to extravasate. Further away, growth inhibition due to soluble factors may become dominant. However, our model has not taken into account circulating lymphocytes or antibodies that may play an important role. Despite its limitations, the overall implication of this work is that therapy targeting a primary tumor can perturb the host immune response in a way that allows increased growth in disseminated disease without altering any of the underlying parameters describing the system.

The full model framework presented here can be translated into more focused systems aimed at addressing specific questions concerning melanoma invasion and treatment. With our model we have demonstrated that disruption of the immune response caused by surgical excision of a primary tumor is a possible mechanism for increased metastasis growth. Following surgery the wound healing response and associated inflammatory response probably also plays a role in cancer recurrence. A more detailed examination of tumor excision with a wound healing cascade could give insight into the importance of immune disruption. The effects of additional treatments such as chemotherapy or radiation therapy on the immune response and metastasis growth should also be investigated. Mathematical modeling may be particularly suited to examining the effect of different treatment schedules. At the least, predictions could be made concerning the efficacy of pre- versus post-operative adjuvant therapy.

Finally, this framework provides an opportunity to investigate the nature and power of the natural selective forces at work driving the evolution of aggressive melanoma tumors. By incorporating multiple cell strains with differential parameter values, we can study the spatial and temporal requirements for successful mutant strain invasion of a pre-existing tumor and how allopathic intervention alters the balance of selective forces in and around the primary tumor.
